# Neuroinfections caused by fungi

**DOI:** 10.1007/s15010-018-1152-2

**Published:** 2018-05-21

**Authors:** Katarzyna Góralska, Joanna Blaszkowska, Magdalena Dzikowiec

**Affiliations:** 1 Department of Biomedicine and Genetics, Medical University of Lodz, Pomorska 251, 92-213 Lodz, Poland; 2 Department of Diagnostics and Treatment of Parasitic Diseases and Mycoses, Medical University of Lodz, Pomorska 251, 92-213 Lodz, Poland

**Keywords:** Fungi, Neuroinfection, Fungal CNS infections, Fungal meningitis, Risk groups, Symptoms

## Abstract

**Background:**

Fungal infections of the central nervous system (FIs-CNS) have become significantly more common over the past 2 decades. Invasion of the CNS largely depends on the immune status of the host and the virulence of the fungal strain. Infections with fungi cause a significant morbidity in immunocompromised hosts, and the involvement of the CNS may lead to fatal consequences.

**Methods:**

One hundred and thirty-five articles on fungal neuroinfection in PubMed, Google Scholar, and Cochrane databases were selected for review using the following search words: “fungi and CNS mycoses”, CNS fungal infections”, “fungal brain infections”, " fungal cerebritis”, fungal meningitis”, “diagnostics of fungal infections”, and “treatment of CNS fungal infections”. All were published in English with the majority in the period 2000–2018. This review focuses on the current knowledge of the epidemiology, clinical presentations, diagnosis, and treatment of selected FIs-CNS.

**Results:**

The FIs-CNS can have various clinical presentations, mainly meningitis, encephalitis, hydrocephalus, cerebral abscesses, and stroke syndromes. The etiologic factors of neuroinfections are yeasts (*Cryptococcus neoformans, Candida* spp., *Trichosporon* spp.), moniliaceous moulds (*Aspergillus* spp., *Fusarium* spp.), Mucoromycetes (*Mucor* spp., *Rhizopus* spp.), dimorphic fungi (*Blastomyces dermatitidis, Coccidioides* spp., *Histoplasma capsulatum*), and dematiaceous fungi (*Cladophialophora bantiana, Exophiala dermatitidis*). Their common route of transmission is inhalation or inoculation from trauma or surgery, with subsequent hematogenous or contiguous spread. As the manifestations of FIs-CNS are often non-specific, their diagnosis is very difficult. A fast identification of the etiological factor of neuroinfection and the application of appropriate therapy are crucial in preventing an often fatal outcome. The choice of effective drug depends on its extent of CNS penetration and spectrum of activity. Pharmaceutical formulations of amphotericin B (AmB) (among others, deoxycholate-AmBd and liposomal L-AmB) have relatively limited distribution in the cerebrospinal fluid (CSF); however, their detectable therapeutic concentrations in the CNS makes them recommended drugs for the treatment of cryptococcal meningoencephalitis (AmBd with flucytosine) and CNS candidiasis (L-AmB) and mucormycosis (L-AmB). Voriconazole, a moderately lipophilic molecule with good CNS penetration, is recommended in the first-line therapy of CNS aspergillosis. Other triazoles, such as posaconazole and itraconazole, with negligible concentrations in the CSF are not considered effective drugs for therapy of CNS fungal neuroinfections. In contrast, clinical data have shown that a novel triazole, isavuconazole, achieved considerable efficacy for the treatment of some fungal neuroinfections. Echinocandins with relatively low or undetectable concentrations in the CSF do not play meaningful role in the treatment of FIs-CNS.

****Conclusion**:**

Although the number of fungal species causing CNS mycosis is increasing, only some possess well-defined treatment standards (e.g., cryptococcal meningitis and CNS aspergillosis). The early diagnosis of fungal infection, accompanied by identification of the etiological factor, is needed to allow the selection of effective therapy in patients with FIs-CNS and limit their high mortality.

## Introduction

While it is estimated that 1.5 million fungal species exist, only about 70,000 have been formally described. Of the described species, 300 may show virulence to humans, and only 10–15% of these could influence the CNS [1, 2]. Clinically relevant fungi being etiological agents of fungal infections of the CNS include yeasts, filamentous fungi, and dimorphic fungi. Yeasts are unicellular organisms and include the cosmopolitan fungal species of genera *Candida* and *Cryptococcus* (except endemic *C. gattii*), and less common fungi such as *Trichosporon* spp. The filamentous fungi, which are characterized by branching hyphae, include moniliaceous (light-colored) moulds with septate hyphae (*Aspergillus* spp., *Fusarium* spp.) and Mucoromycetes with non-septate hyphae (*Rhizopus, Rhizomucor*, and *Mucor*). They have worldwide distribution and are common causes of fungal CNS infections [3–5]. Pigmented moulds (darkly pigmented) are seen less common and include species which are considered as true neurotropic fungi such as *Cladophialophora bantiana* (mainly in India), *Exophiala dermatitidis* (encountered worldwide, common in East Asia), *Rhinocladiella mackenziei* (mainly in Middle East), and *Verruconis gallopava* (syn. *Ochroconis gallopava*, worldwide). The dimorphic fungi with two morphological stages: mould in environment (25 °C) and yeast in tissue (37 °C) (*Blastomyces, Histoplasma, Coccidioides*, and *Paracoccidioides*) are geographically restricted to specific endemic areas (see part Dimorphic fungi) [3–5].

The incidence of fungal infections is increasing every year, with greater numbers of infections noted among patients belonging to high-risk groups such as HIV-infected persons and AIDS patients, transplant recipients, and immunosuppressed patients treated with chemotherapeutics or corticosteroids, as well as those suffering from haematological disorders and chronically ill patients [3, 4, 6, 7]. Certain conditions may predispose the patient to the development of a specific etiological agent: disease/treatment-associated and genetic factors (prolonged antibiotic therapy, neutropenia, steroid therapy, transplantation, chronic granulomatous disease, CARD9 deficiency, neurosurgery, contaminated devices, and prematurity in infants—*Candida*; diabetic ketoacidosis, necrotic burns, renal failure, and intravenous drug use—Mucoromycetes; contact with birds—*Cryptococcus* and *Histoplasma*; deferoxamine therapy and iron overload—Mucoromycetes [3, 5].

However, some fungi, such as *Cryptococcus, Coccidioides*, and *Histoplasma*, can also cause infection in immunocompetent patients. In USA, it was estimated that invasive mycoses caused by *Candida* spp. are responsible for 72 to 228 infections per million population annually, while *Cryptococcus neoformans* is responsible for 30–66 infections and *Aspergillu*s spp., 12–34 infections [8]. The most common CNS fungal infection worldwide is cryptococcal meningoencephalitis. Disseminated mycosis is often associated with CNS involvement. It is estimated that between 67 and 84% of patients with invasive cryptococcosis develop CNS mycosis, 3–64% develop invasive candidiasis, 40% blastomycosis, 25% disseminated coccidioidomycosis, and 5–20% disseminated histoplasmosis, while 12% develop mucormycosis and 4–6% invasive aspergillosis [3]. It is important to note that examples of FIs-CNS differ in clinical manifestation, depending on type of etiological factor (Table 1).

**Table 1:** Clinical syndromes of neuroinvasions caused by fungi

Clinical syndromes	Genus/class
Meningitis	*Cryptococcus* *Coccidioides* *Exserohilum* *Candida* *Histoplasma*
Meningoencephalitis	*Cryptococcus* *Coccidioides* *Candida*
Brain abscess	*Aspergillus* *Candida* Mucoromycetes *Blastomyces* *Coccidioides* *Histoplasma*
Rhino-cerebral	*Mucor* *Rhizopus* *Absidia* *Rhizomucor* *Syncephalastrum*
Skull-base syndromes	*Aspergillus*
Stroke/infarction	Mucoromycetes *Aspergillus*
Disseminated	Mucoromycetes *Candida* *Cryptococcus* *Aspergillus* *Coccidioides*

Depending on the size of the fungal forms developing in the human body, such as blastospores or hyphae, various forms of CNS lesion may exist. For example, *Blastomyces, Histoplasma, Coccidioides, Paracoccidioides, Cryptococcus*, and *Candida* enter the capillaries and subarachnoid spaces, causing meningitis and subpial ischemic lesions, *Candida* enter the blood vessels and cause local necrotic lesions, while *Aspergillus, Cladosporium*, and Mucoromycetes penetrate large blood vessels and can cause strokes [9, 10].

## Fungal interaction with the BBB and CNS invasion

The pathogenesis of FIs-CNS is not yet fully understood. Penetration of pathogen across the blood–brain barrier (BBB) is an essential step for CNS invasion. The circulating pathogens in the blood must first be arrested in the brain microvasculature and then transmigrate into the brain parenchyma across the blood–brain barrier BBB. Three mechanisms have been described for pathogens to cross the BBB: transcellular migration, paracellular migration, and the Trojan Horse Mechanism [11, 12]. Those mechanisms are best understood for *Cryptococcus* and *Candida. C. neoformans* mechanically arrested in the brain vasculature can cross the BBB by both direct and indirect mechanisms [13]. Direct way include BBB passage through transcytosis of endothelial cells [13, 14], while indirect include transport inside of phagocytes as Trojan Horse Mechanism. In addition, paracellular passage of the *C. neoformans* between endothelial cells has been also suggested [13]. To trigger the translocation processes, mainly through paracellular and transcellular mechanisms, interactions between pathogen protein molecules and BBB are necessary. Recent studies indicate that using transcellular mechanism by *C. neoformans* in brain microvascular endothelial cells (BMECs) requires protein kinase C-alpha activation. The CPS1 gene is required for *C. neoformans* adherence to the surface protein CD44 of human BMECs [15]. The dissemination of pathogen into the brain is controlled by Isc1 gene encoding an enzyme that hydrolyzes inositol. Recently, Huang et al. [16] shown that invasion of *Cryptococcus neoformans* into human BMECs is mediated through the lipid rafts- endocytic pathway via the intracellular kinase-DYRK3. Adherence of *C. albicans* to extracellular matrix is facilitated by fibronectin, laminin, and vitronectin. It was demonstrated that *C. albicans* invasion of brain endothelial cells is mediated by the fungal invasions Als3 and Ssa1 [17]. Als3 binds to the gp96 heat shock protein that is expressed specifically on brain endothelium, promoting endothelial transcytosis by the fungus [18]. Trojan horse pathway starts with an infection of a phagocyte in the periphery. Once internalized, the pathogen may actively manipulate the phagocyte to promote migration towards the brain. The infected phagocyte reaches the brain and adheres to the luminal side of brain capillaries and crosses the BBB, either paracellularly or transcellularly [19].

In states of reduced immunity, the BBB permeability increases, which facilitates the penetration of fungi into the brain.The pathogens get to the brain parenchyma and proliferate causing brain inflammation. As the pathogenic factors have to overcome the effective barriers surrounding the brain, invasions are mostly associated with immunocompromised states. Hence, the activation of nerve cells by fungal cells and the expression of immune-enhancing and immune-suppressing cytokines and chemokines play a determining role in pathogenesis of FIs-CNS [11]. CNS involvement takes place as the blood–brain barrier, and cerebral and subarachnoid spaces are crossed by the invading fungi [12]. This process is favoured by various blood–brain barrier disruptions, such as trauma, surgery, or activation of microglia and cytokines: TNF-α disrupts the tight junctions of the barrier [12]. The rate and extent of infection are influenced by the virulence of the fungus and the activity of the host immune system [12]. T cells, microglia, astrocytes, and endothelial cells play important roles in preventing infection by inhibiting fungal growth through the production of cytokines (INF-γ, TNF-α, IL-1β, IL-6, and IL-12), chemokines, nitric oxide and superoxide anion, and by the expression of MHC I and II molecules [1, 7, 11, 12]. TLR-2, 4, and 9 are responsible for the recognition of fungal antigens: polysaccharide capsule (*C. neoformans*), pseudohyphae (*C. albicans*), or conidia (*Aspergillus* spp.); however TLR-2, Dectin-1, and CR-3 are responsible for the recognition of carbohydrates, such as mannose and β-glucans, present on the surface of *A. fumigatus* and *C. albicans* [12]. Fungal pathogen-associated molecular patterns are connected with carbohydrates (chitin, mannoproteins, phospholipomannan, and β-glucans) in the cell wall which may allow fungal infections to be controlled by enabling the activation of microglia yielding pro-lymphatic and humoral responses. The activation of microglia cells depends on the presence of opsonins and T cells [12].

## Etiological factors of FIs-CNS

### *Cryptococcus* spp.

The etiologic agents of cryptococcosis are Basidiomycetes of the genus *Cryptococcus*. Two species in particular, viz.,* C. neoformans* (three serotypes: A, D, and AD) and *C. gattii* (two serotypes: B and C), are considered dangerous to humans. Two varieties of *C. neoformans* have been distinguished: *C. neoformans* var. *neoformans* and *C. neoformans* var. *grubii* [2, 3, 20]. The fungal virulence factors include melanin production and the presence of a polysaccharide capsule. In vitro studies indicate greater induction of expression of HLA class II genes, and hence greater virulence, in human astrocytes by *C. neoformans* than *C. gattii* [21]. *C. neoformans* has a worldwide distribution associated with the presence of pigeon droppings, while *C. gattii* is mostly found on eucalyptus and other tropical and subtropical trees in Australia, Papua New Guinea, parts of Africa, Mexico, and Southern California, Pacific northwest region of the USA, and British Columbia [2, 22].

The blastospores of *Cryptococcus* spp. are phagocytised by pulmonary macrophages, which allows them to survive intracellularly and spread by increasing their resistance to oxidative stress, one of the mechanisms of neutralizing pathogens. The survival of phagocytised cells depends on the activity of laccase: an enzyme taking part in melanin synthesis in *Cryptococcus* sp. [13]. Primary infection involves the lungs; however, the infection typically then spreads further, mainly through continuity or the hematogen and lymphoid pathways, potentially resulting in the involvement of internal organs and the CNS. Infection is also possible through the skin if fungal cells enter a wound [3, 22]. Penetration into the CNS occurs by transport inside macrophages (Trojan Horse Mechanism), transcytosis, or paracellular infiltration. The migration of macrophages infected by fungal cells into the brain causes inflammation, which results in increased permeability of the blood–brain barrier. The most likely reason for the survival of *Cryptococcus* sp. cells in the presence of human microglia cells is the reduced production of NO [12, 13].

*C. neoformans* is the most common opportunistic CNS fungal pathogen observed in HIV-positive patients. Davis et al. [23] observed CNS involvement in the course of generalized cryptococcosis in 82.2% of HIV-infected patients, 48.1% of transplant patients, and 27.6% of immunocompetent subjects. In contrast, a prospective study of patients with cryptococcosis in seven hospitals (USA—four, New Zealand—one, Australia—one, and Taiwan—one) found invasion of the brain in 44% of non-immunosuppressed persons, in 33% of immunosuppressed patients and in only 8% of those infected with HIV, with the mortality rate being greatest for the non-immunosuppressed patients; these findings suggest that, in non-immunosuppressed patients, CNS invasion is associated with poorer prognosis [24]. It is estimated that meningoencephalitis caused by *C. neoformans* affects 1 million people annually [25].

One of the most common forms of CNS cryptococcosis is meningitis, a condition which is considered to be an indicator disease in the development of fulminant AIDS, and which can affect up to 40% of patients [3]. Cryptococcal meningitis is currently characterized by a high mortality rate in patients with AIDS (between 15% and 39%) [26]. Approximately 223,100 cases of cryptococcal meningitis were diagnosed worldwide among people living with HIV/AIDS in 2014: 162,500 in Sub-Saharan Africa, 43,200 in the Asia-Pacific region, 5300 in Latin America, 4400 in Europe, 3300 in North Africa and the Middle East, 3000 in North America, and 1400 in the Caribbean. Every year, 181,100 people die of cryptococcal meningitis worldwide, with 75.04% of them in sub-Saharan Africa [26].

Cryptococcal meningitis is characterized by head and neck pain [20]. As many as 20% of patients show altered mental status, less often convulsions and strokes. Unusual manifestations include paralysis of the lower limbs and weak dorsiflexion, photophobia, nausea and vomiting, transient word winding, disorientation, neologisms, and paraphasic errors [20]. Additional skin lesions may be present in 5% of patients and secondary cerebral infarction in 4% [20].

A very rare manifestation of cerebral cryptococcosis is as intracranial mass lesions, defined as cryptococcoma. Cryptococcoma may be indicated by the presence of a ring-shaped enhancement of mass lesions, with or without cystic changes, under MRI. The most common locations include the basal ganglia, cerebellum or parietal lobe, with the lesions being found more rarely in the intraspinal area and pons [27, 28]. Most symptoms are similar to cryptococcal meningitis: headaches, nausea, vomiting, confusion and high body temperature, convulsions, depression, mental changes, drowsiness, confusion, polyuria, and cranial nerve tract deficiency [27, 28].

### *Candida* spp.

The most commonly identified opportunistic fungi in humans are the Ascomycetes of the genus *Candida*. In immunocompetent individuals, they may be part of the natural microbiota of the oral cavity, gastrointestinal tract, and urogenital tract. In a situation where the natural defence mechanisms of the organism are compromised, *Candida* spp. may over-proliferate and lead to the development of invasive candidiasis [3, 6, 29]. Immunosuppressed patients, and those with neutropenia, diabetes, AIDS or extensive wounds (burns and operations) are particularly susceptible to candidiasis, as well as premature infants, those recovering from transplantation, or patients who have undergone catheterisation [30]. Disseminated candidiasis may result in the infection of the lungs, respiratory system, and digestive tract [31]. Patients with a significantly impaired immune system can develop a disseminated form characterized by the presence of fungemia. Involvement of the CNS is mainly observed in patients who develop disseminated candidiasis or who have undergone neurological surgery [3, 32–35]. A characteristic feature of *Candida* sp. is the formation of a biofilm, which protects yeast cells from microglial activity (phagocytosis, cytokines, NO) [12]. It is estimated that 6% of patients with disseminated candidiasis have unrecognized neuroinfection [36].

Meningoencephalitis is the most common clinical manifestation of neurocandidiasis; the other clinical presentation includes endophthalmitis, multiple cerebral abscesses with ring enhancement or nodular enhancing lesions, vasculitis, intraventricular fungus balls, hydrocephalus, calcifications, and cranial neuropathies, with rare cases demonstrating stroke syndromes [3, 29, 34, 36, 37]. Mortality in the course of invasive candidiasis is estimated to range from 10 to 70%, increasing to 90% in cases of CNS involvement [29, 35, 38].

Infants constitute a particularly high-risk group, with 25% of those with birth weight below 1500 g developing invasive candidiasis. Risk factors for neonates include Apgar scores below 5, prolonged antibiotic use, catheterization, total parental nutrition, parental lipids, endotracheal intubation, surgery, and prolonged hospitalization [39]. CNS involvement has been found in 66% of newborns with disseminated candidiasis and 80% with candidal endocarditis. The mortality of invasive candidiasis in newborns is 30%. Neurological changes include meningitis, micro-and macro-abscesses, granuloma, mycotic aneurysm, vasculitis, haemorrhagic necrosis, and demyelization [37, 39].

It is important to note that *Candida albicans* is commonly isolated from both immunocompromised and immunocompetent patients, but its prevalence has begun to decrease over the past decade, while the frequency of non-*C. albicans* species has gradually increased [32]. It should be noted that the incidence of *Candida* spp. in the CNS has increased from 6 to 17% in neurosurgical patients [6]. In patients with underlying malignant diseases or HSCT *C. albicans* was responsible for 33% of neuroinfections, with non-*Candida* species accounting for 77%; *C. parapsilosis* was most frequently observed, while *C. krusei, C. glabrata, C. tropicalis*, and *C. guilliermondii* were also isolated in individual cases. Multi-species infections are also described [3, 32, 40].

### *Aspergillus* spp.

The fungi of the genus *Aspergillus*, phylum Ascomycota, are widely distributed is throughout the environment. Some species are phytopathogens, and numerous representatives may be etiologic agents of human mycosis [41]. Aspergillosis most commonly occurs in the lungs; however, 40% of patients develop extrapulmonary infections [42, 43]. Between 10 and 20% of patients demonstrate CNS involvement in the course of invasive aspergillosis [44–46]. According to Shamim et al. and Mohamadi et al., high prevalence of invasion within the CNS is seen in tropical and subtropical areas, such as India, Sudan, Pakistan, Saudi Arabia, Turkey, and USA [41, 43]. The most important risk factors for brain infection are neutropenia and corticosteroid use. In addition, immunocompromised, organ transplant recipients, oncological, haematological, and AIDS patients also are at higher risk, as are individuals with autoimmune disorders, pulmonary diseases, diabetes, or maxillofacial infections, as well as patients with cytomegalovirus or various forms of cranial trauma [41, 43, 44]. Cases of aspergillosis that affect non-immunosuppressed individuals are more often presented in the world literature [43, 45, 47]. CNS involvement is associated with a high mortality rate of 90–100% in immunosuppressed patients and 40–80% in immunocompetent individuals [41, 43, 44]. Treatment with voriconazole improves patient survival by up to 35–47% [48, 49].

Fungal infections by *Aspergillus* in humans can be caused as many as 200 species, which are classed into 19 disease-causing subtypes. Changes in the CNS are most commonly caused by *Aspergillus fumigatus* in patients with immunosuppression, and by *A. flavus* in immunocompetent individuals. The species *A. niger, A. terreus, A. nidulans, A. clavatus, A. glaucus, A. oryzae, A. sydowii, A. versicolor*, and *A. ustus* are also observed, but less frequently [41, 44, 50]. In the case of aspergillosis, it is extremely important to identify the species, because some are characterized by increased resistance to antifungal drugs, for example *A. terreus* [49].

The progression of neuroaspergillosis occurs by development of infection within the ear, sinusitis, and mastoiditis, and the dissemination of fungal elements to the brain [44]. The most common sources of infection are by the sinuses (27.6%) of patients; 60–70% in immunocompetent patients [44], or during lung diseases (26.8%), although infection is rarely observed through the ear canal (2.4%) [44, 47, 49]. Fungi can also penetrate directly into the CNS as a result of cranial trauma (2.4%) after injury or neurosurgery. Direct hematogenous spread is also possible due to the affinity of the *Aspergillus* genus to blood vessels. In 30–50% of patients with sinusitis and CNS involvement, damage to the skull bones can also be observed. In the course of neuroaspergillosis, the invasion gates are unknown to as many as 20–30% of non-immunosuppressed individuals [41, 44]. A very important virulence factor of *Aspergillus* sp. is its ability to produce mycotoxins which can damage and kill microglia, astrocytes, and neurones. These mycotoxins inhibit phagocytosis and increase conidial resistance to opsonisation [12].

Neuroaspergillosis in immunosuppressed patients may lead to cerebral infarction, haemorrhage, encephalomalacia, cerebrovascular accident (stroke), haemorrhagic or mycotic aneurysm, multiple or isolated abscess, and meningitis [44, 47, 51–53]. Immunocompetent individuals may experience multiple alterations of the cranial nerves, cavernous sinus syndrome, orbital sinus invasion syndrome, granuloma, brain abscess, and meningitis [44, 45, 47].

Clinical manifestations include neurological deficits (50%), fever (45–50%), headache in patients with brain abscess (60%), oedema papillae nervi optici (25%), and seizures (25%). Less common symptoms include stiff neck, cranial or somatic nerve weakness, paresthesia, altered mental status, spinal cord compression, and very rare lethargy and coma [44, 45, 49, 52]. X-ray and CT may show single or multiple aspergilloma in the brain, but this changes show no characteristic images and that is why the mentioned methods are not helpful in the diagnostics [54].

### Mucoromycetes

Fungi of the phylum Zygomycota often occur in the natural environment, especially in areas with high humidity. Species of the genera *Mucor, Rhizopus, Rhizomucor*, and *Lichtheimia* belonging to the order Mucorales are considered pathogenic [55–57]. It is estimated that the annual incidence of mucormycosis is 1.7 per 1 million inhabitants [56]. Infection is caused by the inhalation of spores present in the air, or by damage to the skin or mucous membranes. Those at higher risk include patients with immunosuppression, particularly those with monocytic and granulocytic deficiencies; these inhibit fungal growth by producing oxidative metabolites and cationic peptide defensins [55, 58]. In the course of the disease, the CNS is often involved. The most common form of invasion is rhino-orbital-cerebral mucormycosis, reported in 33–49% of patients. Other forms of the disease are less frequent: cutaneous (10–16%), pulmonary (10–11%), disseminated (6–12%), and gastrointestinal (2–11%) [56]. Neutropenia or glucocorticoid therapy is considered risk factor of rhino-cerebral or/and pulmonary infection, while the patients with diabetes usually develop rhino-cerebral mucormycosis [5].

Of the patients with rhino-orbital-cerebral mucormycosis, the largest group (58.9–86.7%) is people with diabetes, especially diabetic ketoacidosis [56, 59–62]. In the course of diabetes, monocyte, macrophage, and neutrophil function are impaired, the inflammatory response is suppressed, and the immune response is delayed. Conversely, increased glucose levels favourably affect fungal growth [12, 59, 61]. Infection of the CNS was also observed in 13.3–22% of patients with haematological disorders, and less often in transplant recipients, patients with gastroenteritis or with renal failure [56, 59, 60]. Mortality in the course of rhino-orbital-cerebral mucormycosis varies between 30–97% depending on the time of diagnosis and progression of lesions [57, 61, 62].

Occupation of maxillary sinus and ethmoid sinus, orbit, and brain is commonly observed in the course of rhino-orbital-cerebral mucormycosis. The most common symptoms include headache, especially in the face, periorbital oedema, often with loss of vision, fever, diplopia, rhinitis, and decreased mental function. Black nasal discharge, crusts, necrosis of the turbine, ulceration, and palatal perforation are also frequently observed. Cerebral infarction with neutrophilic infiltration and angioinvasion has been noted [56, 59–62]. Histopathology examination of direct and fixed preparations of available biological materials (cerebrospinal fluid, biopsy specimens, surgical resection specimens, and autopsy material) reveals thick, hyaline, branched, non-septate hyphae. Unfortunately, cultures are only possible in 33–61% of cases [56, 57, 59]. The most commonly identified species is *Rhizopus arrhizus* (syn. *R. oryzae*), recorded in approximately 37% of cases, with *Rhizopus microsporus, Rhizomucor pusillus, Apophysomyces variabilis*, and *Lichtheimia ramosa* being less commonly found [57, 59, 63].

A worrying rise in infections in immunocompetent individuals has been seen in recent years. Bala et al. [57] found no risk factors in 24% of patients with mucormycosis. *Apophysomyces elegans* was often identified in infections in immunocompetent patients. CNS occupancy was most often the result of traumatic inoculation or soil contamination. This species causes rhino-orbital-cerebral mucormycosis with symptoms similar to those caused by other Mucoromycetes, however, much more common in the tropical zone [64, 65].

### Dematiaceous fungi

The dematiaceous Ascomycetes fungi are characterized by dark-colored hyphae, due to the presence of melanin. They are found in the soil in all climates. In humans, they can cause phaeohyphomycosis with a number of distinct clinical manifestations, including cutaneous and subcutaneous infection, paranasal sinus infection, and disseminated or cerebral infections. Melanin is considered the most important virulence factor, as it interferes with microglial cells [12]. The most common etiological factor for phaeohyphomycosis is *Cladophialophora bantiana*, recorded in 48% of patients; the second most common is *Rhinocladiella mackenziei* (syn. *Ramichloridium mackenzei*) (13%). *Ochroconis gallopavum, Fonsecaea monophora, Exophiala castellania, Exophiala dermatitidis, Bipolaris spicifera, Nodulisporium* spp., *Aletrnaria* spp., *Rhinocladiella atrovirens*, and others were noted less frequently [66–68].

A number of dematiaceous fungi exhibit neurotropism and can cause primary infection in the CNS. The majority of cases were recorded from North America, followed by Asia, the Middle East, and Europe, and only isolated cases have been reported from Africa, South America, and Australia [66]. As many as 52% of the patients had no risk factors, while 37% of the cases were associated with immunosuppression, 15% were transplant recipients, 10% involved patients with hematologic malignancy and solid tumors, and only 4% had neutropenia. Some patients displayed more than one risk factor [66]. The literature data show that this mycosis is three times more common in men [66, 69, 70]. In addition, 87% of patients demonstrated a brain abscess and 71% had single lesions. Granulomatous inflammation, meningitis, myelitis, or encephalitis was seen less commonly. Mortality was estimated at approximately 74% in immunocompetent patients and 71% in immunosuppressed patients.

Most patients were farmers or residents of agricultural regions, and this could be due to the fact that the main source of *Cladophialophora bantiana* infections is soil. Worldwide, the infection is three times more common in men than women, while India presents a much higher ratio of 14:1 [69]. The most common clinical form is brain abscess, with meningitis observed less frequently. Mortality is above 70%. The predisposing factors are not yet fully understood, most cases involving immunocompetent individuals. The primary site of infection is the lungs, from where the fungus can disseminate to the brain via the hematogenous route. Non-specific clinical manifestations include hemiparesis, headache, and fever. In some patients, the lesions that develop in the brain can be similar to tuberculosis granulomas [69–73].

*Rhinocladiella mackenziei* has not so far been isolated from the environment, but only from biological materials from patients with cerebral phaeohyphomycosis. Most infections have concerned immunocompetent persons, and a few patients with diabetes mellitus, haematological malignancies, chronic liver disease, renal transplants, and systemic lupus erythematous. Until recently, the occurrence of this species was restricted to regions of Saudi Arabia, Kuwait, Syria, and Afghanistan, but its range has since extended to India and Pakistan. Mortality in the course of infection is almost 100% [74, 75].

The fungi of the genus *Fonsecaea* are found in soil and plant material in tropical areas, mainly in South America, Africa, and Japan. Based on the sequence of genes encoding rRNA, three morphologically similar species have been described as potential pathogenic agents for humans: *F. nubica, F. pedrosoi*, and *F. monophora* [76]. They are associated with chromoblastomycosis, but *F. monophora* has a higher affinity for the nervous system [77]. CNS involvement usually occurs by direct or hematogenous spread, with simultaneous occupation of the cerebral cortex. The most important symptoms of CNS invasion are black, necrotic brain tissue, black pus, and black cerebrospinal fluid. X-ray images show ring and hyperdense or hypodense lesions [67, 78, 79].

### Dimorphic fungi

The most common pathogenic dimorphic fungus is *Histoplasma capsulatum*, which endemically occurs in the United States, Latin America, Africa, and Asia. It is considered to be the most common fungal pathogen of the respiratory system. Infection affects about 40 million people in the world [80]. Immunosuppressed patients, AIDS patients, transplant recipients, corticosteroid and tumor necrosis factor antagonists, and patients with ventriculoperitoneal shunts [81] are most at risk. Histoplasmosis develops in 3–5% of people experiencing immunosuppression, among whom 90% develop the disseminated form; it has been found that 10–20% of patients experience CNS involvement, with mortality of 20–40% [80, 82]. Due to the high prevalence of CNS histoplasmosis in AIDS patients, Nyalakonda et al. [83] suggest the differentiation of cases of CNS lesions in AIDS patients in histoplasmosis direction. The increasing rate of *Histoplasma capsulatum* infections in non-endemic areas is of concern, as is the increasing frequency of histoplasmosis with infections of the CNS to 20–30% of patients, in immunocompetent persons [82, 84–86].

The pathogenesis of CNS infection with dimorphic fungi is still largely unknown: The process is partially described only for *Histoplasma capsulatum*. In most cases, histoplasmosis starts with spore inhalation and pulmonary infection. Yeast-phase cells are then phagocytosed by macrophages and intracellular forms spread through the host organism [80]. Up to 50–90% of patients are asymptomatic, while 80% of symptomatic patients do not require treatment. In many cases, the infection appears after a few years, and CNS occupancy has been observed after a long latency or after relapse with delayed manifestation [80, 84, 85]. CNS lesions are not specific and may resemble infections with other etiologic agents. One of the most characteristic symptoms of CNS involvement is hydrocephalus, as well as acute or chronic meningitis. Lowered consciousness was reported in 28.8% of patients, headache in 24%, cranial nerve deficiency in 19.2%, and seizures in 13.5% [80, 84]. Some single and multiple ring-enhancing lesions, short-term memory loss, and cognitive impairment have also been reported [87, 88]. However, the differentiation of histoplasmosis is difficult due to the difficulty in obtaining the culture and the possibility of cross-reactivity with *Blastomyces dermatitidis, Paracoccidioides brasiliensis*, and *Penicillium marneffei* in serological diagnostics, allowing to detect specific antibodies (in serum) and antigens (in serum, CSF, and urine) [82, 89].

Coccidioides is an endemic genus of fungi in Mexico and South America which often causes meningitis in humans. In endemic areas, the risk of infection is over 15% [90]. There are two species within the genus: *C. immitis* is more widespread in California, while *C. posadasii* is common in Texas, Central and South America. Currently, mortality in coccidioidomycosis is less than 30% [90]. The biggest threat to *C. immitis* infection concerns immunosuppressed patients, including those with HIV, or those with diabetes or who are receiving chronic steroid treatment. Even short-term stays in endemic areas may promote neuroinfections [3]. CNS involvement manifests itself as meningitis: headache, fever, altered mental status, personality changes, nausea, and focal neurological deficiency. In 50% of cases, meningismus is observed. Hydrocephalus (in 30–50% of patients), cerebral, and vasculitic infarction (in 15–20% of patients) may occur in the course of illness or as a distal effect after treatment, while spinal arachnoiditis, cerebral abscesses, and mass lesions occur as complications of infection [3, 90].

In Africa and Central America, the dimorphic species *Blastomyces dermatitidis* is endemic. CNS involvement occurs rarely in the course of blastomycosis; it is usually associated with disseminated infection and affects between 5%—in immunocompetent, up to 40%—in AIDS patients. Symptoms within the CNS include brain abscess, epidural abscess, meningitis, and mass lesions [3].

Another dimorphic fungi is *Talaromyces marneffei* (earlier *Penicillium marneffei)*, occurring in Southeast Asia (Southern China, Northeastern India). *T. marneffei* most commonly occurs in immunosuppressed patients (> 80%), especially HIV-infected patients whose CD4 count is 50 cells/mm^2^. In the course of disseminated penicilliosis, fungi were isolated from bone marrow (100%), skin lesions (90%), blood (76%), and lymph nodes (34%); CNS involvement was found only in a few cases [91, 92]. The observed symptoms were non-specific and included altered mental status, confusion, agitation, depressed consciousness, and fever [92].

### Unusual fungi causing CNS mycosis

#### Yeasts

In very rare cases, brain invasions have been attributed to the genus *Trichosporon*, phylum Basidiomycota. Yeasts of the genus *Trichosporon* usually cause fungal infections of the skin and subcutaneous tissue, most often in patients with neutropenia and haematological malignancies. Until 2016, world literature describes only eight cases of CNS infection. *T. asahii* was the most frequent etiological factor of brain lesion, and *T. cutaneum* and *T. beigelii* were identified only once. Patients were found to have chronic meningitis, brain abscess, and hydrocephalus. Intraventricular fungal ball was observed in one case. In cases of trichosporonosis, patients have elevated levels of galactomannan antigen, which is characteristic for infections caused by *Aspergillus* sp. [93, 94].

#### Moulds

In recent years, there has been a rapid increase in the number of infections caused by fungi of the genus *Fusarium*. The reason for invasive infections with *Fusarium* species is likely the profound immunosuppression in affected hosts, because invasive infections with this fungus are extremely rare in immunocompetent individuals [12, 95]. *Fusarium* has a greater tendency to invade patients with haematological malignancies, transplant recipients and prolonged neutropenia due to its affinity for blood vessels. *Fusarium* species possess several virulence factors, including the ability to produce mycotoxins, including trichothecenes and fumonisins, which suppress humoral and cellular immunity. Fumonisin B1, the fungus most common mycotoxin, has been associated with cases of cerebral fungal invasion leading to neuronal axon demyelination. Fumonisin B1 is cytotoxic to microglia causing the accumulation of phospholipids in the cell membrane and altering cellular respiration by impairing mitochondrial function [12]. Among filamentous fungi, *Fusarium* is the second most frequently observed invader in patients with immunosuppression after *Aspergillus* sp. [96–98]. The mortality in fusariosis range 50–80%. *Fusarium* sp. may cause cutaneous, sino-pulmonary and disseminated infections, onychomycosis, osteomyelitis, fungus, and in lens user keratitis. Several CNS infection cases have also been reported, with the majority being diagnosed in the last several years. Patients show multiple brain abscess and meningitis [96, 97]. Of the 69 *Fusarium* species that can cause mycosis in humans, *F. solani, F. oxysporum*, and *F. moniliforme* dominate [96]. In most cases, the fungi involved in CNS infection were identified only to the genus, and two species of *F. solani* and *F. oxysporum* were diagnosed three times [97].

Fungi of the genus *Penicillium*, belonging to Ascomycota, often occur in the environment, develop in dead organic matter, and may also cause plant diseases. The genus includes about 200 species. Due to the small size and large number of spores produced, they may be associated with the occurrence of allergic symptoms, and on vary rare occasions, may infect humans [91, 99]. The species of the genus *Penicillium* are rarely isolated from the human body, and the described cases of invasion mainly concerned immunocompetent people. *P. commune, P. chrysogenum, P. decumbens, P. citrinum*, and *P. brevicompactum* were sporadically reported as etiological agents of penicilliosis. The most common clinical manifestations were superficial mycosis, onychomycosis, dermatitis, keratitis, conjunctivitis, and otomycosis, while lung infection, endocarditis, urinary tract infection, endophthalmitis, oesophagitis, and intracranial infections also occurred but more rarely. Neuropenicilliosis proceeded with mycotic cerebral aneurysym, multiple brain oedema or brain abscess. *P. commune* (isolated from brain autopsy) and *P. chrysogenum* (isolated from CSF and brain biopsy) were found to be associated with CNS infection, and in most cases, the identification was made only to the genus [91, 99, 100].

*Scedosporium apiospermum* (teleomorph *Pseudallescheria boydii*), from phylum Ascomycota, is widely distributed in the environment, particularly in soil, water, and decaying organic matter. It is the most common cause of chronic subcutaneous gain white mycetoma (Madura foot). In addition, it can cause endophthalmitis, pneumonia, endocarditis, lung abscess, disseminated disease, and CNS involvement [101, 102]. Symptoms of CNS infection include solitary mass or multiple brain abscess (76.9%), and meningitis and mycotic aneurysms. Mortality in the course of infection is 76% [101–103]. Patients with insulin-dependent diabetes mellitus, lupus erythematosus, transplant recipients, patients with leukemia and other cancers, and near-drowning victims are most at risk. Men are twice more likely to be infected than women, and the majority of patients are children and adolescents. The most common cause of CNS infection is contact with contaminated water [101, 102, 104]. A few cases of CNS infection with *S. apiospermum* after near-drowning have been described in the world literature [105, 106]. In addition, endophthalmitis due to *S. prolificans* (syn. *Lomentospora prolificans*) has been reported on rare occasions [107].

## Diagnosis of FIs-CNS

In accordance with EORTC/MSG criteria, the diagnosis of invasive fungal infections is made on the basis of a combined interpretation of risk factors, clinical symptoms, and imaging results. Symptoms of CNS invasion are in most cases not very specific and include headache, fever, convulsions, weakness, progressive confusion, changed mental status, and/or focal neurological deficits among others [6, 7]. In addition, the image of mycosis in CT, MRI imaging may resemble changes caused by other pathogenic factors. For candidiasis or cryptococcosis, CT imaging is usually negative, and focal lesions are seen in the course of infections with mould fungi. Non-specific focal lesions, edema, or haemorrhagic lesions are found in the MRI image. In addition, therefore, CT and MRI techniques can only serve as additional aids in the diagnosis of FIs-CNS [3, 7]. These imaging techniques can help localize the lesions, but tissue samples are still needed for objective diagnosis, especially in cases of fungal abscesses.

The diagnosis of invasive mycosis requires biopsy of the involved tissue, followed by culture and histopathology of clinical samples; however, CNS biopsies are regarded as too risky in severely ill patients, especially in populations of haematological patients with low platelet counts or neutropenia. Biopsy allows CNS specimens to be obtained, including those of the brain, meninges and cerebrospinal fluid (CSF) or ventricular fluid [108]. Methods based on optical brighteners (Calcofluor or Blankophor) allow direct examination and have high sensitivity and specificity for detecting fungal elements. Biopsy material stained with hematoxylin and eosin (HE), particularly with Gomori methenamine silver (GMS) or periodic acid Schiff (PAS), is of great importance in the diagnosis of neuroinfections [109–111]. Cell type depends on the pathogen causing neuroinfection: lymphocytic pleocytosis is associated with *Cryptococcus neoformans*; neutrophils or monocytic predominance with *Candida* spp.; neutrophilic predominance with *Aspergillus* spp.; eosinophilia with *Coccidioides* spp. [3]. Diagnosis should be confirmed by culture, if possible, and serological tests of blood and cerebrospinal fluid. The cultures can be performed on Czapek-Dox and Sabouraud media, but their effectiveness varies depending on the etiological agent. Peripheral blood cultures are most likely to be useful when the etiologic agent is *Candida* spp. It should be noted that *Fusarium* and *Scedosporium* species can easily be recovered from the bloodstream in patients with disseminated infections, which is rare for mould infections. Other fungi, such as *Histoplasma capsulatum* and *Cryptococcus neoformans*, can occasionally be isolated from blood cultures [112].

Serological tests are mainly based on ELISA, EIA, and indirect hemagglutination techniques, and in recent years, immunofluorescence techniques have developed significantly [3, 7, 113]. The sensitivity of antibody assays varies between 38% and 92% depending on the species, while the detection of fungal cell wall components (β-glucans, galactomannan, mannan, and chitin) in serum or CSF ranges from 64 to 90% [37, 114]. Fungal antigens (e.g., mannan from *Candida* spp., galactomannan from *Aspergillus* spp., and galactoxylomannan from *Cryptococcus* spp.) can be detected with commercially available kits. The detection of cryptococcal capsular polysaccharide antigen in serum or CSF by the latex agglutination test is an established method to diagnose cryptococcosis [115], while serum assays for galactomannan are recommended for diagnosing invasive aspergillosis [116]. However, it should be emphasized that as galactomannan antigens can also be expressed by *Fusarium*, their positive detection in the serum or CSF does not constitute a definitive diagnosis of CNS aspergillosis [5]. In addition, (1–3)-β-D-glucan being a cell wall constituent of *Candida* and *Aspergillus* species, and several other fungi, is not specific serum marker for invasive candidiasis or aspergillosis [37, 111]. Although serological tests based on the detection of specific antibodies or antigens in body fluids (urine, serum) are commonly used in the diagnostics of CNS histoplasmosis, these methods show variable cross-reactions with other dimorphic fungi including *Blastomyces* spp. and *Coccidioides* spp. [116]. A recent study found the CSF antigen test to be effective in the diagnosis of coccidioidal meningitis (sensitivity 93%, specificity 100%) [117].

More accurate confirmation of diagnoses, and identification of fungi, could be achieved by polymerase chain reaction (PCR) assays. Unfortunately, there are still no standardized and validated detection methods based on PCR; most PCR protocols have been developed for the diagnosis of invasive fungal infections, mainly for *Aspergillus* and *Candida* species [108, 116, 118–121]. The retrospective analysis of CSF samples from patients with suspected CNS-invasive aspergillosis found the nested PCR assay to have high sensitivity [118]. PCR positivity in CSF was observed for 8/8 proven/probable, in 4/22 possible and in 2/25 patients without invasion yielding sensitivity and specificity values of 100% and 93%, respectively. PCR techniques performed on cerebrospinal fluid samples may well become the new gold standard for the diagnosis of CNS infection, especially for patients whose clinical condition does not allow invasive diagnostic procedures; however, validation is first needed. In addition, other molecular diagnostic tools for the rapid detection of fungi directly from CSF, such as fluorescence in situ hybridization (FISH), have proved promising in clinical trials but still need to undergo standardization before clinical use [113, 122].

## Treatment of FIs-CNS

Fungal neuroinfections are characterized by higher mortality rates and poorer prognosis than viral and bacterial infections, and parasitic invasions. Rapid diagnosis and the use of appropriate therapy are crucial in helping prevent an often fatal outcome. The choice of antifungal therapy depends on the fungistatic and fungicidal action of drug. The fungal cell membrane or wall components (ergosterol, chitin, and β-glucans) are major targets of the main groups of antifungal agents in current use, with the exception of flucytosine (antimetabolic effects) [1, 3]. The mode of action of selected antifungal drugs and their spectrum of activity against fungal species is summarized in Table 2. Amphotericin B deoxycholate (AmBd) is highly toxic and has poor CNS penetration, but is an effective treatment for cryptococcal meningoencephalitis, in combination with flucytosine, and neuroinfections caused by other fungi which are not susceptible to agents with good CNS penetration (e.g., voriconazole); in these cases lipid formulations of amphotericin B (L-AmB) should be preferred [123–125]. Treatment recommendations (IDSA, ESCMID, and ECMM) for the most common fungal CNS infections are presented in Table 3 [37, 110, 111, 125–130]. Among antifungal drugs, voriconazole, fluconazole, and flucytosine readily penetrate into the CNS, but itraconazole and posaconazole only penetrate to a minor degree [131]. Voriconazole is recommended as primary therapy for CNS aspergillosis, while liposomal amphotericin B (L-AmB) are reserved for intolerant or refractory patients [111, 132, 133]. Clinical data indicate that isavuconazole shows satisfactory activity in invasive aspergillosis [134] and disseminated mucormycosis with location in CNS [135].

**Table 2:** Principle properties of selected antifungal drugs used for treatment of central nervous system infections [1, 3, 39, 54, 80, 123, 124, 130, 133]

Antifungal agent	Selected pharmacokinetics properties	Mode of action	Spectrum of activity
Amphotericin B (Polyene antibiotic) Amphotericin B formulations Deoxycholate—AmBd Lipid complex—ABLC Colloidal dispersion—ABCD Liposomal—L-AmB	Concentration-dependent pharmacokinetics—for all formulations Plasma protein binding—95–99%—for all formulations *T*_1/2_ of AmBd of 15–27 h *T*_1/2_ of ABLC of ~ 24 h *T*_1/2_ of L-AmB of 12–24 h *T*_1/2_ of ABCD of ~ 18 h Vd (L/kg) of AmBd—0.5–2.0 Vd (L/kg) of L-AmB—0.05–2.2	Binding and interaction with ergosterol and destabilization of fungal cell membrane; increase in cell membrane permeability for mono- and divalent cations, which leads to cell death	*Mucor* *Absidia* *Aspergillus* *Cryptococcus* *Candida* *Histoplasma* *Blastomyces* *Coccidioides* *Paracoccidioides* *Sporothrix*
Fluconazole (Triazole)	Dose-proportional pharmacokinetics (except in patients with renal impairment) Plasma protein binding—12% *T*_*1*/*2*_ of 24–30 h Vd (L/kg)—0.7	Inhibition of cytochrome P-450 14 α-lanosterol demethylase; accumulation of lanosterol leading to disorders in the cell membrane	*Candida* (except *C. glabrata* and *C. krusei*) *Cryptococcus* *Histoplasma* *Blastomyces* *Coccidioides*
Itraconazole (Triazole)	Concentration-dependent pharmacokinetics Plasma protein binding 99.8% *T*_1/2_ of 34 h Vd (L/kg)—11	Inhibition of ergosterol synthesis in the fungal cell membrane like other triazoles	*Aspergillus* *Cryptococcus* *Candida* *Histoplasma* *Paracoccidioides* *Blastomyces* *Sporothrix*
Voriconazole (Triazole)	Concentration-dependent pharmacokinetics Plasma protein binding—58% *T*_1/2_ of ~ 6 h Vd (L/kg)—45	The mechanism of action on fungi is similar for fluconazole	*Candida* *Aspergillus* *Fusarium* *Scedosporium*
Posaconazole (Triazole)	Dose-proportional pharmacokinetics Plasma protein binding > 98% T_1/2_ of 20–31 h Vd (L/kg)—20 (oral suspension) Vd (L/kg)—5 (tablet formulation) Vd (L/kg)—3.7 (intravenous)	Fungicidal action similar to other triazoles	*Aspergillus* *Candida* *Coccidioides* *Fusarium* *Rhizomucor* *Mucor* *Rhizopus*
Isavuconazole (Triazole)	Dose-proportional pharmacokinetics Plasma protein binding > 98% *T*_*1*/*2*_ of 80–120 h Vd (L/kg)—65	The mechanism of action on fungi is similar for fluconazole	*Candida Aspergillus* *Mucor* *Rhizopus* *Rhizomucor* *Fusarium* *Sporothrix*
Flucytosine/5-FC/(Nucleoside)	Dose-proportional pharmacokinetics (except in patients with renal impairment) Plasma protein binding—5% *T*_*1*/*2*_ of 3–5 h Vd (L/kg)—0.4–0.8	Weakening of nucleic acid synthesis by formation of toxic, fluorine pyrimidine antimetabolites It enters the fungal cell by cytosine permease where it is deaminated to the active form of 5-fluorocytosine, which weakens the synthesis of DNA and RNA	*Cryptococcus* *Candida* *Cladophialophora Fonsecaea* *Phialophora*

*Vd* apparent volume of distribution, *T*_*1/2*_ half-life

**Table 3:** Treatment recommendations (IDSA, ESCMID, and ECMM) for fungal CNS infections

Fungal CNS infection	First-line therapy		References R^*^
	Adults	Children	
Cryptococcal meningoencephalitis	Initial therapy (for the non–HIV-infected and non-transplant patients): AmBd (0.7–1.0 mg/kg/day IV) plus flucytosine (100 mg/kg/day) ≥ 4 weeks AmBd (0.7–1.0 mg/kg/day IV) ≥ 6 weeks—for flucytosine-intolerant patients L-AmB (3–4 mg/kg/day) or ABLC (5 mg/kg/day IV) combined with flucytosine ≥ 4 weeks—for AmBd-intolerant patients Consolidation therapy: fluconazole (400–800 mg /day) ≥ 8 weeks Maintenance therapy: fluconazole (200 mg/day) for 6–12 months	Initial therapy: AmBd (1 mg/kg/day IV) plus flucytosine (100 mg/kg/day orally in 4 divided doses) for 2 weeks L-AmB (5 mg/kg/day) or ABLC (5 mg/kg/day) for AmBb-intolerant patients Consolidation therapy: fluconazole (10–12 mg/kg /day orally) for 8 weeks Maintenance therapy: fluconazole (6 mg/kg/day orally) for 6–12 months	[125] IDSA
Cerebral cryptococcomas	Initial therapy: AmBd (0.7–1 mg/kg/day IV), or L-AmB (3–4 mg/kg/day IV), or ABLC (5 mg/kg/day IV) plus flucytosine (100 mg/kg/day orally in 4 divided doses) ≥ 6 weeks Consolidation and maintenance therapy: fluconazole (400–800 mg/day orally) for 6–18 months Adjunctive therapies Corticosteroids for mass effect and surrounding edema Surgery: for large (≥ 3 cm lesion), accessible lesions with mass	No recommendation	[125] IDSA
Candidiasis	Initial therapy: L-AmB (5 mg/kg/day), with or without oral flucytosine (25 mg/kg 4 times daily) for several weeks Step-down therapy: fluconazole, 400–800 mg (6–12 mg/kg) daily. Therapy should continue until all signs and symptoms and CSF and radiological abnormalities have resolved	Initial therapy for the neonatal patients: AmBd (1 mg/kg/day IV) or L-AmB (5 mg/kg/day IV) for several weeks Step-down therapy: fluconazole (12 mg/kg/day). Therapy should continue until all signs, symptoms, and CSF and radiological abnormalities, have resolved	[37] *IDSA*
Aspergillosis	Voriconazole: loading dose, 6 mg/kg IV every 12 h for 1 day, maintenance dose 4 mg/kg IV every 12 h L-AmB (3–5 mg/kg/day IV) for intolerant or refractory to voriconazole Therapy should continue until all signs and symptoms and CSF and radiological abnormalities have resolved	Voriconazole: loading dose, 9 mg/kg IV every 12 h for 1 day; maintenance dose, 8 mg/kg IV every 12 h L-AmB (3–5 mg/kg/day IV) for intolerant or refractory to voriconazole Treatment duration is determined on a case-by-case basis	[111] *IDSA*
Mucormycosis	L-AmB (10 mg/kg/day IV), initial 28 days The duration of antifungal treatment should be determined on an individual basis, usually continues for at least 6–8 weeks	L-AmB (5–10 mg/kg/day IV). Treatment duration is determined on a case-by-case basis	[110] ESCMID and ECMM
Hyalohyphomycosis *Fusarium* species and *Scedosporium apiospermum* infection	Voriconazole: loading dose (6 mg/kg IV every 12 h for 1 day) maintenance dose (4 mg/kg IV every 12 h) for long-term	Voriconazole: 4 mg/kg IV every 12 h (13–18 years old) and 8 mg/kg IV every 12 h (2–12 years old)	[129] ESCMID and ECMM
Histoplasmosis	Initial therapy: L-AmB (5.0 mg/kg/day for a total of 175 mg/kg given over 4–6 weeks) Step-down therapy: itraconazole (200 mg 2 or 3 times daily) for at least 1 year and until resolution of CSF abnormalities, including *Histoplasma* antigen levels	Initial therapy: AmBd (1 mg/kg/day IV) for 2 weeks Step-down therapy: itraconazole (5.0–10.0 mg/kg/day in 2 divided doses), not to exceed 400 mg daily; for at least 1 year	[126] *IDSA*
Blastomycosis	Initial therapy: L-AmB (5 mg/kg/day) over 4–6 weeks Step-down therapy: fluconazole (800 mg/day), itraconazole (200 mg 2 or 3 times per day), or voriconazole (200–400 mg twice per day) for at least 12 months and until resolution of CSF abnormalities	Initial therapy: AmBd (0.7–1.0 mg/kg/ day), or L-AmB (3–5 mg/kg/day) Step-down therapy: oral itraconazole, 10 mg/kg/ day (up to 400 mg/day) for a total of 12 months	[127] *IDSA*
Coccidioidal meningitis	Fluconazole (400–1200 mg /day orally) for patients with normal renal function and without substantial renal impairment; lifelong treatment In patients who clinically fail initial therapy with fluconazole, higher doses are a first option, and second—change of therapy to another orally administered azole, or to initiate intrathecal AmBd therapy	Fluconazole (12 mg/kg/day IV) or ABLC (3–5 mg/kg/day IV), lifelong treatment	[29, 128] IDSA
Cerebral phaeohyphomycosis	No strong recommendation for antifungal treatment When surgery (complete excision) is not possible voriconazole (400 mg) or posaconazole (800 mg) or combination therapy including a triazole plus an echinocandin plus flucytosine are proposed	No recommendation	[130] ESCMID and ECMM

*AmBd* Amphotericin B deoxycholate, *L-AmB* Liposomal amphotericin B, *ABLC* Amphotericin B lipid complex, *IV* intravenous, R* recommendation,

In addition to pharmacological treatment, surgical removal of lesions is also possible. In most cases, a combination of surgical intervention and antifungal therapy increases the survival rate of patients with FIs-CNS [1, 3]. It has been shown that neurosurgery is associated with an improved outcome in patients treated with voriconazole for CNS fungal infections [48]. Surgical intervention can be used in cases of focal or localized superficial cortico-subcortical lesions (such as abscesses and granulomas) in the non-eloquent areas of the brain, while invasive multifocal lesions, deep cerebral, and/or brain stem lesions involving large parts of the brain, and major vascular invasions are not an indication for using surgical therapies.

## Summary

Fungal infections of the central nervous system (FIs-CNS) are rare but pose a threat to life of patients. They are still a significant challenge for diagnosis and treatment. Their prevalence spans a wide array of hosts including immunosuppressed and immunocompetent individuals, mainly hospitalized persons and patients undergoing neurosurgical procedures. *Cryptococcus neoformans, Aspergillus* spp. and *Rhizopus* spp. remain the most common pathogens responsible for neuroinvasions. Recently, the number of newly detected fungal species in the CNS has been increasing, which requires the use of more advanced diagnostic methods to establish an etiology of emerging mycoses. Because the manifestations of FIs-CNS are often non-specific, diagnosis of the infection is very difficult. The clinical picture may mimic other CNS infectious diseases, especially tubercular meningitis, and therefore, precise diagnosis is needed. The routine diagnosis includes direct microscopic examination of clinical samples, histopathology, culture, and serology. The use of PCR-based assays still needs to undergo standardization. Although the radiological characteristics of FIs-CNS are often non-specific, some neurological changes caused by the presence of these pathogens can be revealed by CT and MRI. The choice of appropriate therapy is crucial in helping prevent the high mortality associated with fungal neuroinfections, and the choice of drug depends on its extent of CNS penetration, mode of action, and spectrum of activity.

## Abbreviations

AIDS: Acquired immunodeficiency syndrome
BBB: Blood–brain barrier
BMECs: Brain microvascular endothelial cells
CARD9: Caspase recruitment domain-containing protein 9
CNS: Central nervous system
CSF: Cerebrospinal fluid
CT: Computed tomography
ECMM: European confederation of medical mycology
EORTC/MSG: The European Organization for Research and Treatment of Cancer/Invasive Fungal Infections
ESCMID: The European Society of Clinical Microbiology and Infectious Diseases
FIs-CNS: Fungal infections of central nervous system
HIV: Human immunodeficiency virus
HLA: Human leukocyte antigens
HSCT: Hematopoietic stem cell transplant
IDSA: Infectious diseases society of America
MRI: Magnetic resonance imaging
PCR: Polymerase chain reaction
rRNA: Ribosomal ribonucleic acid
